# Species-specific outcomes in wasp sting challenges during venom immunotherapy and their respective follow-up 

**DOI:** 10.5414/ALX02653E

**Published:** 2026-09-25

**Authors:** Eva Oppel, Felix V. Weber, Benjamin Kendziora, Bernhard G. Weiss, Paul Severin Wiggenhauser, Friedrich Ihler, Maya Bertlich, Nora Aszodi-Pump, Jennifer Lee Spiegel, Lars E. French, Saskia Freytag, Mattis Bertlich

**Affiliations:** 1 Department of Dermatology and Allergy, LMU University Hospital, LMU Munich,; 2 Department of Dermatology, Thalkirchner Street Hospital, Munich Municipial Hospital Group, Munich,; 3 Institute for Health Services Research in Dermatology and Nursing (IVDP), University Medical Center Hamburg-Eppendorf, University of Hamburg, Hamburg,; 4 Department of Otorhinolaryngology, Head and Neck Surgery,; 5 Division of Hand, Plastic and Aesthetic Surgery, LMU University Hospital, LMU Munich, Munich,; 6 Department of Otorhinolaryngology, Head and Neck Surgery, Greifswald University Medicine, Greifswald,; 7 Department of Dermatology, University Hospital Bonn, Bonn,; 8 Department of Otorhinolaryngology, University Hospital, Tübingen, Germany,; 9 Dr. Philip Frost, Department of Dermatology and Cutaneous Surgery, University of Miami Miller School of Medicine, Miami, FL, USA, and; 10 The Walter and Eliza Hall Institute of Medical Research and Department of Medical Biology, University of Melbourne, Melbourne, VIC, Australia

**Keywords:** wasp allergy, sting challenge, insect species, hymenoptera

## Abstract

Background: Insect venom allergy is a major cause of life-threatening anaphylaxis, with venom immunotherapy (VIT) being the gold standard for prevention. Sting challenges (SC) are used to assess immune tolerance and VIT efficacy, but factors influencing outcomes remain unclear. This study examined whether the stinging insect species affects SC outcomes during VIT and evaluated outcomes during long-term follow-up. Materials and methods: A retrospective analysis of 1,163 patients undergoing SC during wasp venom VIT was performed. The stinging species was entomologically confirmed. Associations between SC tolerance and species, skin prick test (SPT) results, venom-specific IgE levels, and treatment duration were analyzed using multivariable logistic regression adjusted for demographics. Follow-up assessed subsequent field stings and systemic reactions. Results: SCs were conducted with *Vespula vulgaris *(n = 479), *Vespula germanica* (n = 615), *Dolichovespula media* (n = 40), and *Dolichovespula saxonica* (n = 29). SC was tolerated without systemic reaction in 1,141 patients (98.1%). *Vespula spp.* were associated with a significantly higher likelihood of tolerance (odds ratio (OR) 7.69, 95% CI 2.66 – 20.43) compared with *Dolichovespula spp.* A decline in SPT positivity occurred after VIT, and conversion from positive to negative at 10 µg/mL showed a trend toward improved sting tolerance (OR 2.26, 95% CI 0.68 – 11.53). During follow-up (mean 53.2 ± 38.5 months), 167 patients experienced field stings; 6 developed systemic reactions, none of whom had been challenged with *Dolichovespula spp*. Conclusion: Species-specific differences influence sting challenge outcomes, suggesting incomplete cross-protection of *Vespula* VIT against *Dolichovespula spp.* Declining SPT reactivity may predict sting tolerance. SCs remain a reliable indicator of post-therapy protection against anaphylaxis.

## Introduction 

Insect venom allergies are among the most frequent causes of life-threatening anaphylaxis in adults [1]. Up to 3% of adults in Europe have reported previous systemic reactions to insect stings [2]. Venom immunotherapy (VIT) has proven to be the gold standard for preventing severe allergic reactions in sensitized individuals and is regarded as a highly effective treatment [3, 4]. However, exact predictions of the degree of anaphylaxis or the efficacy of VIT remains difficult [5, 6]. A component of VIT evaluation involves sting challenges using live insects, which serve as the most reliable method to assess immune tolerance and therapy efficacy [7, 8]. Despite the widespread use of VIT, there is still considerable debate about the application of sting challenges [9]. This is partly due to numerous questions regarding factors that influence sting challenge outcomes which remain unanswered. One such factor is the role of the stinging insect species in determining the success or failure of VIT. 

In central Europe, the majority of clinically significant sting reactions are caused by honeybees (*Apis mellifera*) and social wasps, particularly *Vespula vulgaris* and *Vespula germanica*. Other species, such as *Dolichovespula spp.*, hornets (*Vespa spp*.) [10], and bumblebees (*Bombus spp*.), are less common triggers of allergic reactions. Importantly, venom proteins from hymenopterans often exhibit strong cross-reactivity [11, 12], yet data on cross-reactivity between the (relatively closely) related *Vespula* and *Dolichovespula* species remain scarce. This may be due to the relatively close phylogenetic relation of the species. To date, no systematic studies have investigated the impact of different insect species on sting challenge results in patients undergoing VIT. 

To address this knowledge gap, we conducted a retrospective study of a large cohort of wasp-allergic patients who underwent sting challenges as part of their VIT evaluation. In this cohort, the stinging insect was entomologically identified, allowing for a detailed analysis of species-specific effects on sting challenge outcomes. This study focused on wasp species that are native to Europe, including *Vespula vulgaris, Vespula germanica, Dolichovespula media, and Dolichovespula saxonica*, all of which also exhibit a broad global distribution (Figures 1, 2, 3). By evaluating this extensive dataset, our primary objective was to determine whether the species of the stinging insect influences sting challenge results and to assess the clinical implications of these findings. 

## Materials and methods 

### Ethics 

The present study was conducted in compliance with the Declaration of Helsinki and national guidelines. It was registered with the appropriate authorities and approved by the Ethics Committee of Ludwig-Maximilians University, Munich, Germany (file no. 23-0785). Due to the entirely retrospective and anonymized nature of the study at hand, the ethics committee waived the need for informed consent. 

### Sting challenge protocol 

Sting challenges were conducted as described previously [7, 13]. After patients gave written consent and physicians ensured that there were no contraindications against undergoing sting challenges, patients were placed on an examination table. A wasp that had previously been collected from the environment by a professional beekeeper was then placed on the shoulder of a patient using a dip net. The location was chosen as this is where the sting is the least painful [14]. After the wasp was positioned on the patient’s skin, gentle physical stimulation was applied to induce a sting. Afterwards, the wasp was removed and if the stinger persisted together with the venom sac, it was left for 5 minutes and then subsequently removed as well. Patients were monitored for 60 minutes and remained inpatients for another night in case of late-onset reactions. Anaphylaxis was graded as previously described by Ring and Messmer [15]. After the sting, the stinging insect was euthanized and submersed in 96% ethanol for preservation. All insects were then examined by a professional entomologist for species determination. As the wasps were typically quite active prior to the sting challenge, a precise – or even approximate – entomological identification was unfortunately not feasible. In patients who developed a systemic reaction during the sting challenge, VIT was continued with an increased maintenance dose, typically 200 µg of *Vespula* venom. 

### Patients 

Only patients who underwent their initial sting challenge and who were 18 years or older at the time of the sting challenge were included in the analysis. Data between 2007 and 2024 were available for analysis and included in the study at hand. Individual datasets were extracted from both electronic and physical patient records, encompassing a wide range of variables. These included patient demographics (age, sex), the type of insect allergy (wasp or hornet, and whether coexisting bee allergies were present), results of skin prick tests (SPTs), serological analyses, and the severity of the initial anaphylactic reaction. Pre-existing medications like ß-blockers or ACE inhibitors were omitted from the analysis, as there is evidence that these do not pose a relevant risk factor for systemic reactions [16, 17]. Patients with elevated baseline serum tryptase levels underwent a full-body dermatological examination by a board-certified dermatologist to assess for clinical signs of mastocytosis. If the clinical findings were suggestive of cutaneous mastocytosis, the diagnosis was confirmed by skin biopsy. If systemic mastocytosis was clinically suspected, patients underwent further diagnostic work-up according to the contemporaneous WHO recommendations, including bone marrow biopsy where indicated. As SPTs were routinely recorded by physicians during VIT initiation but performed by nurses before sting challenge, results were only categorized as positive or negative. SPTs were routinely performed at concentrations of 1, 10, 100, and 300 µg/mL following routine clinical procedures. SPTs were performed exclusively with standardized commercial allergen extracts from ALK-Abelló (Hørsholm, Denmark) following routine clinical procedures. Among the tested concentrations, 10 µg/mL showed the most frequent change in SPT reactivity between VIT initiation and sting challenge and was therefore chosen for the regression analysis. Additionally, information on prior venom-specific VIT was collected, including dosage, duration, and the patient’s response to the sting challenge. All patients received commercially available *Vespula* venom preparations according to routine clinical practice. Maintenance injections were administered according to the respective manufacturer’s recommendations. During the study period, different licensed therapeutic allergen preparations were used depending on product availability. Only *Vespula* VIT solutions were used as *Vespula* is currently the only genus with approved products for VIT in Germany. Therefore, even patients who were later stung by different species had been treated with *Vespula* venom extract, reflecting routine clinical practice. The cross-reactivity between these two genera was assumed to provide sufficient protection. Patients were followed up annually after their respective sting challenges. During these follow-ups, records were made of any subsequent field stings or repeated sting challenges, whether systemic reactions occurred, and the patient’s perception of the stinging insect species. Only reactions that led to a clinical consequence (i.e., an increase in dosage or the reinitiation of immunotherapy) and were at least °I according to Ring and Messmer were considered as such. All patient data was anonymized irreversibly during the data collection process to ensure confidentiality. 

### Laboratory analysis 

All allergy-related diagnostic measurements were performed in-house at the Department of Dermatology and Allergy, LMU University Hospital. Venom-specific IgE levels were determined using the ImmunoCAP system (Thermo Fisher Scientific, Uppsala, Sweden) according to the manufacturer’s instructions. *Dolichvespula spp.* venom IgE was not routinely determined. Baseline serum tryptase concentrations were measured using the ImmunoCAP tryptase assay (Thermo Fisher Scientific, Uppsala, Sweden). Wheal reactions were assessed according to national guidelines by measuring the largest wheal diameter in millimeters, with a diameter of ≥ 3 mm considered positive. 

### Statistics 

For descriptive statistics, mean values with standard deviation were calculated for continuous variables and absolute and percentage frequencies for categorical variables. IgE values below the detection limit (< 0.35 kU/L) were assigned a value of 0.00 kU/L, while IgE values exceeding the upper detection limit (> 100 kU/L) were capped at 100 kU/L. 

To assess associations between patient and treatment characteristics and sting challenge tolerance, a multivariable logistic regression model was applied. Given the relatively low number of outcome events, a penalized likelihood approach using Firth’s correction was employed to reduce small-sample bias and to obtain more reliable effect estimates. 

Sting challenge tolerance was included as the dependent variable. The following variables were entered as predictors: wasp species used for the sting challenge (*Vespula spp.* vs. *Dolichovespula spp*.), age (per 10-year increase), sex, baseline serum tryptase (log-transformed), duration of VIT (years), change in SPT reactivity to wasp venom at 10 µg/mL (categorized as positive-to-negative and negative-to-positive), change in specific IgE to wasp venom (per 10 kU/L), maintenance dose of wasp VIT (100 vs. 200 µg), and additional VIT with bee venom (yes vs. no). Odds ratios (ORs) with corresponding 95% confidence intervals (CIs) were reported as measures of association. 

In the follow-up analysis, a χ^2^-test was used to evaluate the association between the species involved in field stings and the occurrence of systemic reactions. Due to the low number of anaphylaxis cases, multivariable regression analysis was not performed for this endpoint. 

The statistical calculations were performed in R (build 4.2.3 for windows, the R project for statistical computing, http://www.r-project.org/) by a professionally trained statistician. 

## Results 

### General characteristics 

Overall, 1.165 patients who underwent their initial sting challenge after wasp VIT were included in the study at hand. Two patients were stung by rare Hymenoptera (1 by *Polistes dominula* and 1 by *Vespa crabro*). Due to their scarcity, these 2 were omitted from the analysis. 

Of the 1,163 patients included in the study, 485 (41.7%) were male and 678 (58.3%) were female. The mean age at the initiation of VIT was 49.2 ± 14.5 years (range: 12 – 86 years), while the mean age at the time of sting challenge was 51.0 ± 14.3 years (range: 18 – 88 years). The average duration of VIT prior to sting challenge was 20.9 ± 19.2 months (range: 1 – 342 months). At the time of sting challenge, 108 patients were also undergoing concomitant VIT with bee venom. Baseline tryptase levels at VIT initiation averaged 6.8 ± 15.6 (range: 1.01 – 140), and cutaneous or systemic mastocytosis was confirmed in 145 patients (12.4%). The general characteristics are summed up in Table 1. 

Of the 1,163 sting challenges performed, 69 involved *Dolichovespula spp*., including 40 with *D. media* and 29 with *D. saxonica*. The remaining 1,094 challenges were conducted with *Vespula spp*., comprising 479 with *V. vulgaris* and 615 with *V. germanica*. Despite undergoing VIT, 22 patients exhibited allergic reactions upon sting challenge. Detailed distributions, including stratification by mastocytosis status, are provided in Table 2. 

Of the 1,163 patients analyzed, 76.0% had a positive SPT at 100 µg/mL before SIT, while 27.5% were positive at 10 µg/mL. After VIT, the proportion of positive SPTs decreased, with only 43.0% remaining positive at 100 µg/mL and 6.5% at 10 µg/mL. Overall, 40.5% of patients converted from positive to negative at 100 µg/mL, while 24.8% converted at 10 µg/mL. Conversely, newly positive reactions were rare, occurring in only 5.6% at 100 µg/mL and 3.6% at 10 µg/mL. Serum IgE levels also declined (mean reduction: 4.2 kU/L). The exact values can be found in Table 3. 

### Associations between patient and treatment characteristics and sting challenge tolerance 

Age was not a significant predictor of a successful outcome (OR: 0.95, 95% CI: 0.68 – 1.34). Interestingly, male sex showed no strong, yet remarkable association with an increased likelihood of a protective effect (OR: 1.70, 95% CI: 0.65 – 5.06). Baseline tryptase levels appeared to have a negative correlation with a favorable outcome, but the effect did not reach statistical significance (OR: 0.65, 95% CI: 0.37 – 1.25). The duration of VIT showed a moderate but nonsignificant association with a successful sting challenge outcome (OR: 1.20, 95% CI: 0.78 – 2.40), suggesting a potential trend toward improved protection with longer treatment duration. An increased maintenance dosage of wasp VIT (200 vs. 100 µg) was not significantly associated with sting tolerance (OR: 1.15, 95% CI: 0.11 – 157.7), although the CI was remarkably wide due to the small number of events. Similarly, concomitant VIT against bee venom showed no significant association with sting challenge outcome (OR: 0.59, 95% CI: 0.16 – 3.20). A conversion from a positive to a negative SPT at 10 µg/mL of wasp venom was associated with increased odds of not reacting to a sting (OR: 2.26, 95% CI: 0.68 – 11.53), though the CI was wide and thus did not reach statistical significance. Conversely, a conversion from negative to positive at 10 µg/mL was not associated with a higher likelihood of reaction (OR: 0.75, 95% CI: 0.16 – 7.29). Changes in specific IgE levels for wasp venom showed no strong predictive value (OR: 0.93, 95% CI: 0.58 – 1.34). However, patients who had undergone a sting challenge with *Vespula spp.* had significantly higher odds of tolerance, with an OR of 7.69 (95% CI: 2.66 – 20.43) compared to *Dolichovespula spp*., indicating a strong correlation between the stinging insect species and the risk of experiencing a systemic reaction despite immunotherapy. 

The exact values can be found in Table 4. 

### Follow-up 

Of the 1,163 sting challenges performed, 545 patients returned for follow-up assessments. The mean follow-up duration was 53.2 ± 38.5 months, ranging from 3 to 215 months. During the follow-up period, 167 of these patients reported experiencing at least one field sting by a wasp. Among them, 14 had previously undergone a sting challenge with *Dolichovespula spp*. (12 with *D. media*, 2 with *D. saxonica*), and 155 had been challenged with *Vespula spp*. (97 with *V. germanica*, 56 with *V. vulgaris*). A total of 6 patients developed a systemic reaction to the field sting. Of these, 5 had previously undergone a sting challenge with *V. germanica*, and 1 with *V. vulgaris*. Out of these 6 patients, 3 were still undergoing VIT, while 3 had completed VIT after 5 years (average of 53.3 months, range 17-98 months since completion of VIT). χ^2^ analysis of the prevalence of systemic reactions among patients who experienced field stings, stratified by the stinging species, yielded a p-value of 0.303. An exact overview can be found in Table 5. 

## Discussion 

The cohort at hand is one of the largest cohorts on Hymenoptera sting challenges that has been published to this date. The risk factors that make a systemic reaction during a sting challenge more likely (age, sex, baseline tryptase, presence of mastocytosis) have been described previously [7]. This is not surprising, as the study by Ruëff et al. [7] was conducted at the same institution. However, the timely overlap (and therefore in patients) is only 3 years. Moreover, for the first time, we observed that patients whose SPT with wasp venom (10 µg/mL) converted from positive to negative between the initiation of VIT and the sting challenge had a noticeably increased likelihood of tolerating the challenge. While this difference did not reach statistical significance, this is the first study to investigate this aspect and may prove significant in future research. Moreover, owing to the low number of systemic reactions, the multivariable regression analyses should be interpreted as exploratory. Although Firth’s penalized likelihood approach was applied to reduce small-sample bias, the limited number of outcome events reduces the precision of several effect estimates. Consequently, these analyses should be regarded as hypothesis-generating rather than confirmatory. 

Although not statistically significant, our data suggest a trend indicating that the likelihood of a successful sting challenge increases with the duration of VIT. To date, no definitive evidence has been established regarding this association. However, findings from the study by Goldberg et al. [8] demonstrated that even within days after initiating VIT, 70 – 80% of patients already tolerated a sting challenge successfully. This is particularly remarkable as this study was conducted with bees, where the chance of treatment failure is significantly elevated. In light of these findings, it seems plausible that longer treatment durations may further enhance the protective effect of VIT, supporting the hypothesis that extended immunotherapy might improve long-term sting tolerance. 

Another remarkable observation in our study was the overall tolerance of sting challenges. It is well established that VIT for wasp venom is very efficient. However, the results were notably more consistent with the findings of Kranert et al. [18] or Schrautzer et al. [19] than with those of Ruëff et al. [7]. This is surprising, given that the latter was conducted at the same institution and even shares a (minor) portion of its cohort with the present study. The most likely explanation for this discrepancy is the increased efficacy of the commercial solutions available for VIT over time. The cohort studied by Kranert et al. [18] and Schrautzer et al. [19] overlaps more closely in time with our study population than the cohort from Ruëff et al. [7]. 

In terms of the influence of the stinging insect, this is one of the first studies that has investigated this aspect of sting challenges. It is striking that the probability of successfully undergoing a sting challenge increases almost 8-fold if the sting is induced by *Vespula spp*.. The only other study that – to the best of our knowledge – has investigated this matter was conducted by Golden et al. [20] at the Johns Hopkins University and University of Pennsylvania. However, there are two major differences: In said study, patients who explicitly did not undergo previous VIT underwent sting challenges. Moreover, the only insects used were *V. germanica* and *V. maculifrons*. While the first is quite common globally, the latter is only known to inhabit northern America. The wasp species in the study at hand show a significantly broader global distribution. Nevertheless, this study and the one by Golden et al. show a remarkable similarity: Both demonstrate a significant, species-specific difference in tolerance of sting challenges. This is remarkable as the phylogenetic relation of *Vespula spp*. and *Dolichovespula spp*. is quite high; the two genera are phylogenetically closely related [21, 22]. Still, this suggests that cross-reactivity among different species may not be as extensive as previously assumed. Taken together, these findings suggest that detailed history taking, thorough serological workup and, where possible, a species-specific approach to VIT might be beneficial, as different vespid species cannot be considered interchangeable in terms of immunogenicity and cross-protection. 

Moreover, this study is the first to investigate a large cohort of sting challenges while also incorporating long-term follow-up. Nearly half of the patients provided follow-up data, with an average follow-up duration exceeding 50 months, ensuring a strong and reliable data foundation. At first glance, the fact that 6 out of 167 patients who experienced a field sting by wasps reacted systemically may seem relatively high. However, 3 of these patients had already completed VIT, with 17 – 98 months having elapsed since completion. Additionally, uncontrolled external factors such as psychological or physical stress, alcohol consumption, or intercurrent infections might have contributed. Finally, the numbers might be attributed to the retrospective nature of the study and its inherent biases. Conversely, when considering the entire cohort of 1,163 sting challenges, only 6 patients (0.5%) exhibited a systemic reaction during the considerable follow-up. This finding supports the diagnostic reliability and safety of sting challenges beyond its well documented effect on quality of life [23, 24], even though it has repeatedly been debated [9, 25, 26]. Although sting challenges with *Dolichovespula spp.* were associated with a higher rate of systemic reactions during the controlled challenge itself, none of these patients experienced a systemic reaction during subsequent field stings in our follow-up. However, the species responsible for field stings could not be entomologically confirmed, as this information relied on patient reports and the insects were not available for taxonomic identification. Therefore, it cannot be excluded that some of these reactions may have been caused by *Vespula spp.*, whose venom had been used for immunotherapy. Given the potentially limited cross-reactivity between *Dolichovespula* and *Vespula* venoms, this might suggest a higher predictive value of sting challenges in this context. However, our dataset does not allow for definitive conclusions in this regard. Consequently, we decided to routinely inform patients about this potential risk and offer the option of an additional sting challenge. To date, no patient has opted for a second sting challenge. 

Finally, the limitations of this study need to be pointed out. First, the general limitations of any retrospective studies apply, as the dataset may be subject to numerous unknown biases or a systematic lack of data. Second, while the follow-up rate of nearly 50% is substantial, recall bias may have influenced patients’ self-reported reactions to subsequent field stings. Third, despite the large sample size, some subgroup analyses – particularly those involving *Dolichovespula spp*. – were limited by the still relatively small number of cases, potentially affecting statistical power. Moreover, no a priori sample size calculation was performed because the study included all eligible sting challenges available during the predefined study period. The small number of systemic reactions limited statistical precision and the ability to detect potentially relevant associations. Therefore, the findings should be considered exploratory and interpreted with caution. Additionally, the relatively high prevalence of mastocytosis in our cohort likely reflects referral bias, as our institution serves as one of the major referral centers for mastocytosis in Germany and therefore treats a disproportionate number of patients with this condition. Moreover, another limitation is that *Dolichovespula* sting challenges were performed after immunotherapy with non–species-identical (*Vespula*) venom, which may introduce confounding: poorer outcomes in the *Dolichovespula* group could be driven by venom mismatch rather than true species-related risk. Accordingly, our findings do not allow separating an intrinsic venom-related effect from a lack of full immunological cross-reactivity between *Vespula* and *Dolichovespula* venoms. 

## Conclusion 

Our findings suggest that sting challenges with *Dolichovespula spp*. exhibit lower protection against *Dolichovespula* sting challenges than against *Vespula* sting challenges, underscoring species-specific differences in venom tolerance and immunogenicity. Additionally, changes in SPT reactivity between VIT initiation and the sting challenge may have clinical significance, though further studies are needed to confirm their predictive value. Long-term follow-up data indicate that sting challenges reliably predict the likelihood of anaphylaxis following subsequent field stings, reinforcing their relevance in patient risk assessment. Given the uncertain (yet seemingly favorable) long-term outcomes, we routinely inform patients who previously underwent a *Dolichovespula spp*. sting challenge about the option of an additional sting challenge, though we do not actively recommend it. 

## Ethics approval and consent to participate 

The study was conducted in accordance with the principles outlined in the Declaration of Helsinki and was registered with the appropriate authorities (Ethics Committee of the Ludwig-Maximilians University of Munich, Germany). General written informed consent was obtained from all participants prior to study inclusion, permitting the use of their data for academic research. As determined by the local Ethics Committee (file no. 23-0785), additional consent for inclusion in this specific analysis was not required due to the retrospective nature of the study. 

## Availability of data and material 

The original, anonymized dataset is available upon reasonable request from the corresponding author. 

## Acknowledgments 

The authors would like to thank Sonja Gassner with her invaluable aid and enthusiasm in this project. The authors would like to point out that the work at hand is part of the doctoral thesis of Felix V. Weber. 

## Authors’ contributions 

Conception of the project: MB; Organization of the project: EO, BGW, SW, FI, MB, LEF, SF, MB; Execution of the project: FVW; Design of the statistical analysis: BK; Execution of the statistical analysis: BK, SF; Review and critique of the statistics: EO, BGW, SW, FI, MB, LEF, NAP, JLS, SF, MB; Writing of the manuscript: MB; Review and critique of the manuscript: EO, FVW, BK, BGW, SW, FI, MB, LEF, NAP, JLS, SF. 

## Funding 

The authors have no relevant funding to disclose. 

## Conflict of interest 

The authors have no relevant conflict of interest to disclose. 

**Figure 1.**
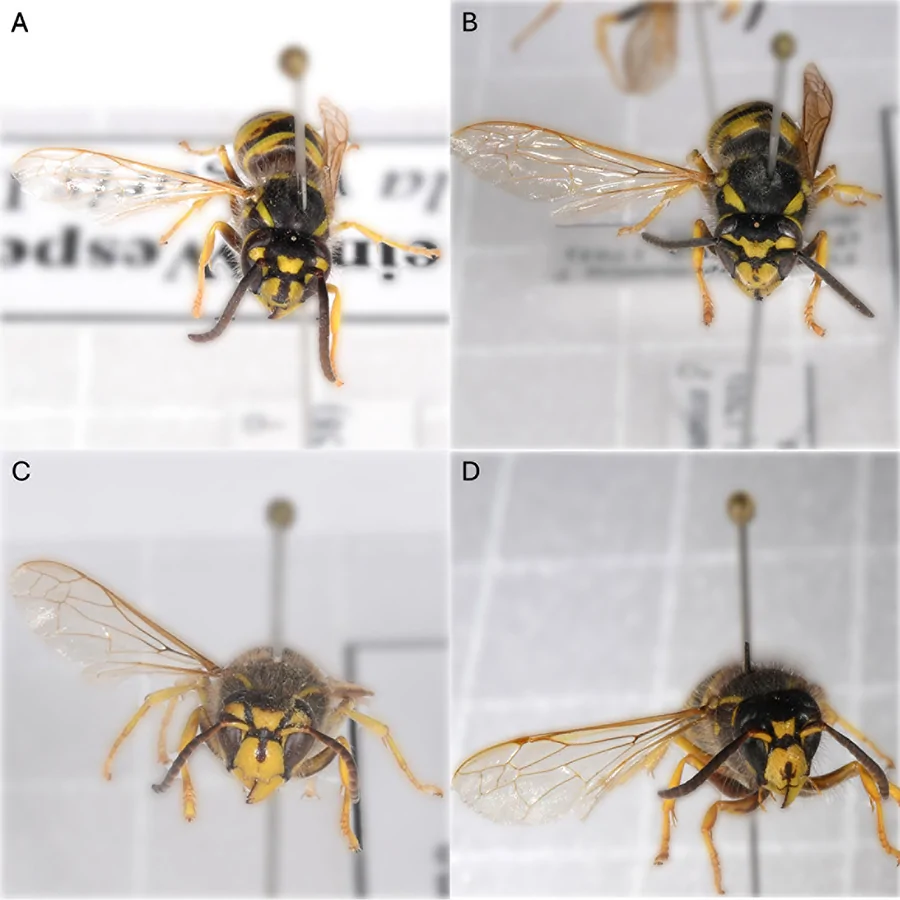
Wasp species used in the study at hand. A: *Vespula vulgaris*; B: *Vespula germanica*; C: *Dolichovespula media;* D: *Dolichovespula saxonica*.

**Figure 2.**
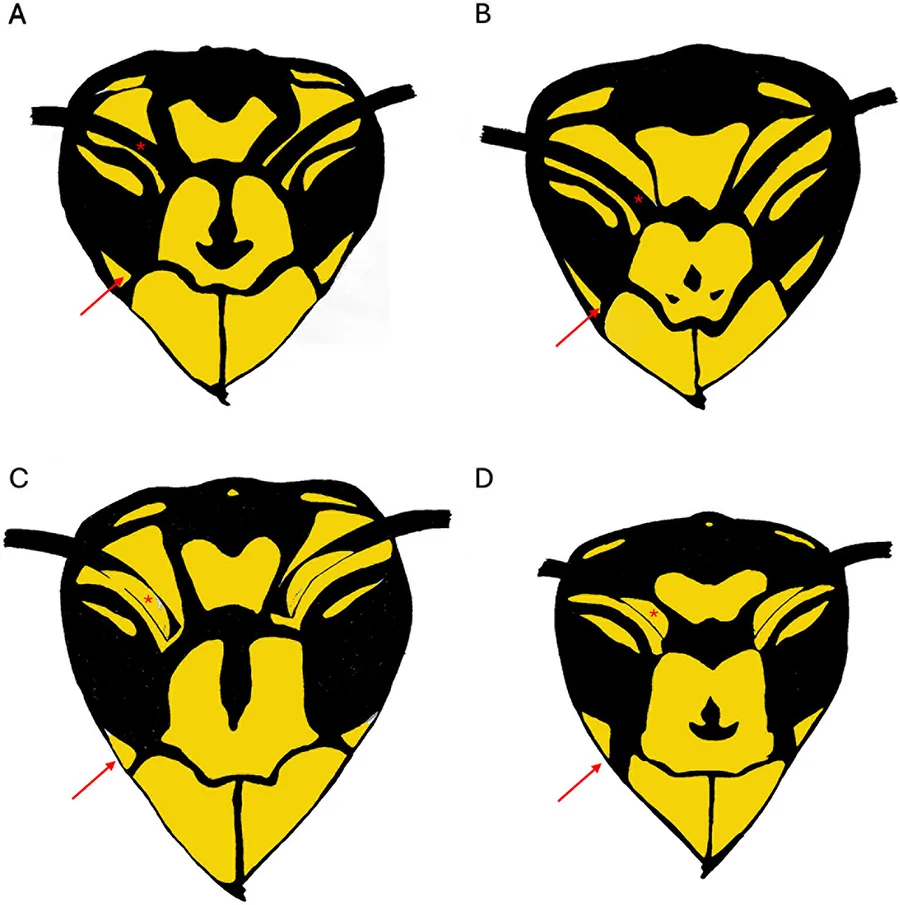
Facial markings distinguishing the wasp species analyzed in this study. A: *Vespula vulgaris,* B: *Vespula germanica,* C: *Dolichovespula media,* D: *Dolichovespula saxonica.* The key distinguishing feature between *Dolichovespula* and *Vespula* species lies in their face shields: In *Vespula spp.*, the mandibles directly contact the eye socket (arrows), whereas in *Dolichovespula spp.*, a distinct gap creates a cheek-like appearance. Additionally, the underside of the antennae is typically black in *Vespula spp.* but yellow in *Dolichovespula spp.* (indicated by *).

**Figure 3.**
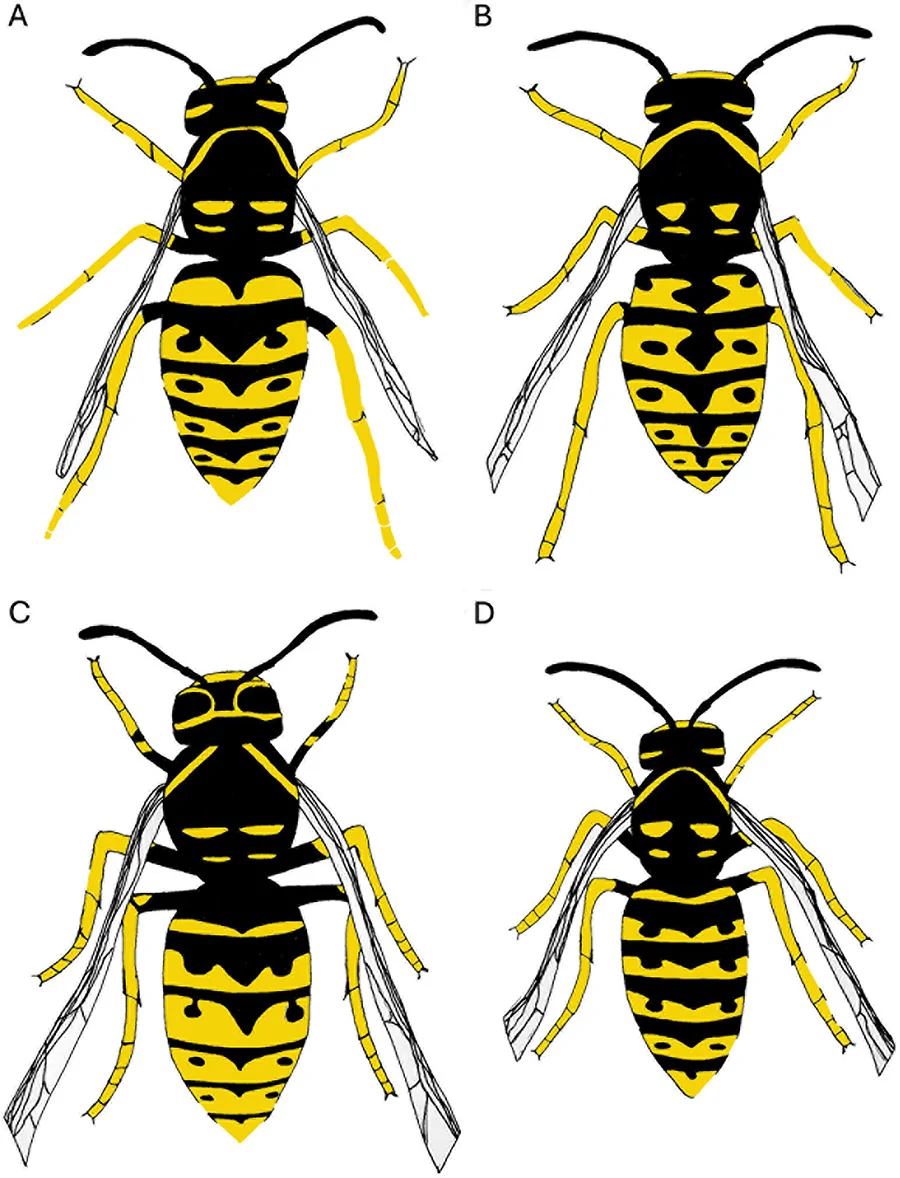
Illustrations of different wasp body shapes of the species analyzed in this study. A: *Vespula vulgaris;* B: *Vespula germanica;* C: *Dolichovespula media;* D: *Dolichovespula.*

**Table 1.** Baseline characteristics of the study population. Continuous variables are expressed as mean ± standard deviation with the corresponding range in brackets, while categorical variables are presented as absolute numbers and percentages.

**Parameter**	**Value**
Total no. of patients	1,163
Sex	
Male	485 (41.7%)
Female	678 (58.3%)
Age at beginning of VIT (years)	49.2 ± 14.5 (12 – 86)
Age at sting challenge (years)	51.0 ± 14.3 (18 – 88)
Duration of VIT before sting challenge (months)	20.9 ± 19.2 (1 – 342)
Concomitant bee venom VIT	108 (9.3%)
Tryptase levels at initiation of VIT (ng/mL)	6.8 ± 15.6 (1.01 – 140)
Confirmed mastocytosis (n)	145 (12.4%)

VIT = venom immunotherapy.

**Table 2.** Distribution of systemic reactions during sting challenges, stratified by mastocytosis status and stinging insect species. Percentages indicate the proportion of systemic reactions within each respective subgroup.

	*Dolichovespula spp.*			*Vespula spp.*			**∑**
	*D. media*	*D. saxonica*	**∑**	*V. vulgaris*	*V. germanica*	**∑**	
No confirmed Mastocytosis	4 / 32 (12.5%)	0 / 27 (0.0%)	4 / 59 (6.8%)	4 / 418 (0.9%)	9 / 540 (1.7%)	13 / 958 (1.4%)	17 / 1.017 (1.7%)
Mastocytosis confirmed	1 / 8 (12.5%)	1 / 2 (50.0%)	2 / 10 (20.0%)	0 / 61 (0.0%)	3 / 75 (4.0%)	3 / 136 (2.2%)	5 / 146 (3.4%)
∑	5 / 40 (10.0%)	1 / 29 (3.4%)	6 / 69 (8.7%)	4 / 479 (0.8%)	12 / 615 (2.0%)	16 / 1.094 (1.5%)	22 / 1.163 (1.9%)

**Table 3.** Changes in skin prick test reactivity and venom-specific IgE levels before and after venom immunotherapy and sting challenge. Means are ± standard deviation, percentages are given in brackets.

		**SPT ** **300 µg**	**SPT ** **100 µg**	**SPT ** **10 µg**	**SPT ** **1 µg**	**IgE**
Before SIT	Positive	371 (31.9%)	884 (76.0%)	320 (27.5%)	47 (4.0%)	10.2 ± 19.1 n = 1,147
	Negative	26 (2.2%)	216 (18.6%)	794 (68.3%)	1,067 (91.8%)	
	n/a	766 (65.9%)	63 (5.4%)	49 (4.2%)	49 (4.2%)	
Before SC	Positive	314 (27.0%)	500 (43.0%)	75 (6.5%)	19 (1.6%)	7.2 ± 12.6 n = 1,098
	Negative	164 (14.1%)	649 (55.8%)	1,074 (92.3%)	1,130 (97.2%)	
	n/a	685 (59.9%)	14 (1.2%)	14 (1.2%)	14 (1.2%)	
Δ	Positive → negative	111 (9.5%)	471 (40.5%)	288 (24.8%)	38 (3.3%)	–4.2 ± 13.2 n = 1,084
	Negative → positive	13 (1.1%)	65 (5.6%)	42 (3.6%)	12 (1.0%)	
	Unchanged	261 (22.4%)	553 (47.6%)	772 (66.4%)	1,052 (90.5%)	
	n/a	778 (66.9%)	74 (6.4%)	61 (5.2%)	61 (5.2%)	

SPT = skin prick test.

**Table 4.** Odds ratios with 95% confidence intervals for numerous variables calculated for the probability of successfully (i.e., without systemic reactions) undergoing sting challenges.

**Variable**	**Odds ratio [95% confidence interval]**
Age (10-year steps)	0.95 [0.68 – 1.34]
Male gender	1.70 [0.65 – 5.06]
Baseline tryptase (logarithmic steps)	0.65 [0.37 – 1.25]
Duration of VIT (in years)	1.20 [0.78 – 2.40]
Increased dosage (<100 µg) of VIT for wasp venom	1.15 [0.11 – 157.7]
Additional VIT against bee venom	0.59 [0.16 – 3.20]
Positive -> negative SPT wasp 10 µg/mL	2.26 [0.68 – 11.53]
Negative -> positive SPT wasp 10 µg/mL	0.75 [0.16 – 7.29]
IgE difference IgE wasp venom (10 kU/L steps)	0.93 [0.58 – 1.34]
Stinging insect *Vespula spp.*	7.69 [2.66 – 20.43]

VIT = venom immunotherapy; SPT = skin prick test.

**Table 5.** Follow-up of patients undergoing sting challenges. Data are presented as absolute numbers (percentage of total in respective category). The percentages in square brackets represent the relative proportion within the respective subgroup.

	**Sting challenges**	**Follow-up**	**Field stings**	**Systemic reaction**
*D. media*	40 (3.4%)	32 (5.9% [80.0%])	12 (7.2% [30.0%])	0 (0.0%)
*D. saxonica*	29 (2.5%)	9 (1.7% [31.0%])	2 (1.2% [6.9%])	0 (0.0%)
*V. vulgaris*	479 (41.2%)	214 (39.3% [45.6%])	97 (58.1% [20.7%])	1 (16.7% [0.2%])
*V. germanica*	615 (52.9%)	290 (53.2% [47.2%])	56 (33.5% [9.1%])	5 (83.3% [0.8%])
∑	1.163 (100.0%)	545 (100.0% [46.9%])	167 (100.0% [14.4%])	6 (100.0% [0.5%])
